# Survival of patients with chronic lymphocytic leukemia before and after the introduction of chemoimmunotherapy in Germany

**DOI:** 10.1038/s41408-021-00556-7

**Published:** 2021-10-29

**Authors:** Hiltraud Kajüter, Ina Wellmann, Laura Khil, Karl-Heinz Jöckel, Can Zhang, Anna-Maria Fink, Michael Hallek, Andreas Stang

**Affiliations:** 1 Cancer Registry of North Rhine-Westphalia, Bochum, Germany; 2 Institute of Medical Informatics, Biometry and Epidemiology, University Hospital Essen, Essen, Germany; 3 Department I of Internal Medicine and Center of Integrated Oncology Aachen Bonn Cologne Duesseldorf and German CLL Study Group, University Hospital Cologne, University of Cologne, Cologne, Germany; 4 School of Public Health, Department of Epidemiology, Boston University, Boston, MA USA

**Keywords:** Prognosis, Epidemiology

## Abstract

Chronic lymphocytic leukemia (CLL) is the most common leukemia of adults in western countries. Therapy is indicated in symptomatic and advanced stages and has changed fundamentally since 2010 when rituximab, an anti-CD20 antibody, has been approved for treatment of CLL. Until then therapy had been based on chemotherapy drugs. This study investigates whether survival in CLL patients improved at the population level after the introduction of combined chemoimmunotherapy. Data from the cancer registry North-Rhine Westphalia was used to calculate relative survival (RS) by applying period analyses. Age-standardized 5-year RS increased from 79% in 1998–2002 (75% in 2003–2007) to 81% in the calendar period 2008–2012 and 88% in 2013–2016 for men and continuously from 71% in 1998–2002 to 92% in 2013–2016 for women. In CLL patients aged 15–69 years 5-year RS increased from 83% to 90% for men and from 82% to 94% for women after adding an anti-CD20-antibody to chemotherapy while in the older age group of 70–79-year-old CLL patients an increase by 20 percentage points was observed. These findings show marked improvements in the survival of CLL patients at the population level subsequently to the approval of anti-CD 20 antibodies like rituximab, ofatumumab or obinutuzumab for CLL treatment.

## Introduction

Chronic lymphocytic leukemia (CLL) is the most common leukemic disease in western countries. The age-standardized incidence rate is four to five cases per 100,000 person-years [1, 2] and about 5500 patients in Germany are newly diagnosed with CLL annually. Men are more often affected than women. CLL is a disease of the elderly with a median age at diagnosis of 70 years. While age-standardized rates remained constant over the last 15 years, the absolute number of cases increased [1]. Hence, healthcare needs will rise further during the next years. CLL is basically diagnosed after routine blood analyses. The diagnosis requires ≥5 × 10^9^/L clonal B- lymphocytes in the peripheral blood and immunophenotyping is mandatory to confirm the CLL with coexpression of CD5 /CD19, CD20, CD23 and low levels of CD20 and CD79b. Expression of either κ or λ immunoglobulin light chains confirms the clonality [3].

The clinical presentation of CLL is diverse and varies between an indolent and a highly aggressive course. Phase 3 trials investigating treatment in early and asymptomatic stages, could not demonstrate an advantage for overall survival [4, 5]. Standard of care for these patients remains a watch-and-wait strategy. Antineoplastic treatment is indicated in advanced stages (Binet C), if symptoms occur (organomegaly, anemia, thrombocytopenia, B-symptoms (fatigue, fever, night sweat, weight loss), or with a lymphocyte doubling time of <6 month [3]. Therapy of CLL has changed fundamentally during the last decade. Until 2009 antineoplastic treatment was based on chemotherapeutic drugs, like chlorambucil or fluradabine as single-agents or fluradabine in combination with cyclophosphamide (FC). Results of the CLL8 study showed a marked improvement of progression-free and overall survival in patients treated with a combination of FC and rituximab, a monoclonal anti-CD20 antibody, in comparison to FC alone [6]. The CLL11 protocol [7] corroborated the notion that survival benefits could be obtained even for older and less-fit patients with marked comorbidities by adding an anti-CD20-antibody (rituximab or obinutuzumab) to chemotherapy (with chlorambucil). The combined chemoimmunotherapy has then rapidly become a standard regime in first-line therapy of CLL patients [8] from its introduction until recently when novel non-cytostatic, targeted agents have led to another paradigm shift in the treatment of CLL, and chemoimmunotherapy has been replaced as standard of care for many but so far not all patients with CLL.

Although controlled clinical trials have shown an improvement in survival achieved by chemoimmunotherapy, firm evidence is still lacking at a population-based level. Until now, there are only two representative population-based studies from Denmark and Sweden that analyzed survival for CLL patients in relation to the introduction of chemoimmunotherapy [9, 10]. The aim of the work presented here was to investigate survival in CLL before and after the introduction of chemoimmunotherapy in a population of 2.6 million people using data of the population-based cancer registry of North Rhine-Westphalia (LKR NRW).

## Material and methods

The Cancer Registry of North Rhine-Westphalia (NRW) covers a population of 18 million people and is the largest cancer registry in Germany. Cancer reporting in NRW is mandatory since 2005. For earlier years from 1993 through 2004, the cancer registry covered a subset of the NRW population located in the north of NRW (administrative district of Münster) covering a population of 2.6 million people. The database of the LKR NRW includes information on patient demographics, tumor diagnosis, tumor characteristics and mortality follow-up.

For this study, we identified newly diagnosed CLL on the basis of the International Classification of Diseases for Oncology (ICD-O) morphology 9823/3 or 9670/3 in the cancer registry file. Patients living in the administrative district of Münster who were diagnosed with CLL during the calendar years 1993–2016 and aged 15–79 years at the time of diagnosis were included. CLL cases that were notified by death certificate only (DCO) were excluded from the analyses. Because of the high proportion of DCO-cases among people aged 80 years or more, we excluded CLL cases aged 80 years or more from the analyses. Mortality follow-up for cancer patients was routinely assessed by the LKR NRW through record linkage with electronic reports on all deceased individuals in NRW obtained from population registration offices.

Age-standardized incidence rates were calculated for the administrative district of Münster and for Germany for the years 2013–2016 using the old European Standard [11].

To estimate cancer specific (net-)survival we calculated 5-year relative survival (RS). RS for a calendar period is defined as the ratio of the observed survival of cancer patients and the expected survival of the general population of the same age, sex and calendar period [12]. Survival time per patient was calculated by the difference between date of diagnosis and death or right censoring whatever came first. RS was calculated using the period approach [13]. Period analysis provides more up-to date survival estimates than the traditional cohort approach, since it exclusively reflects the survival experience of patients within a most recent calendar period, for which mortality follow-up is available. This is achieved by left truncation of observations at the beginning of this period, in addition to right censoring at its end [14]. Therefore, with the period approach changes in the prognosis of cancer patients can be detected timely. Expected survival was estimated by the Ederer II method [15, 16]. We used life tables of the administrative district of Münster stratified by age-, sex- and calendar year. RS was estimated for the calendar periods 1998–2002, 2003–2007, 2008–2012 and 2013–2016. Five-year RS was age-standardized according to the International Cancer Survival Standard (ICSS) [17]. We also computed age-specific RS for the age-groups 15–69 years and 70–79 years. All calculations were done with SAS, version 9.4. RS was estimated by applying the period macro published by Brenner et al. [14].

## Results

During the years 1993 to 2016 in total 3175 patients (1805 men, 1370 women) were registered with CLL in the administrative district of Münster. The median age at diagnosis was 69 for men and 73 for women. After restricting the analyses to patients aged 15–79 years and exclusion of DCO-cases, 2327 cases (1412 men, 915 women) remained for relative survival analyses. The proportion of DCO-cases that had to be excluded was 7.5% for men and 6.1 % for women. The age-standardized incidence rate for 2013 to 2016 was 5.0/100.000 for men and 2.7/100.000 for women in the administrative district of Münster (Germany: 5.1/100.000 men, 2.7/100.000 women) (Table 1).

**Table 1:** Characteristics of the study population for the period 1993–2016 (administrative district of Münster, North Rhine-Westphalia, Germany).

	Study period 1993–2016		
	Men	Women	Total
Number of cases, *n* (%)			
Total (incl. DCO)	1805 (56.9)	1370 (43.1)	3175
Study population (age 15–79 years; excl. DCO)	1412 (60.7)	915 (39.3)	2327
DCO cases among patients aged 15–79 years (%)	115 (7.5)	59 (6.1)	174 (7.0)
Median age at diagnosis (all ages; incl. DCO)	69.4	73.4	70.9
Median age at diagnosis (study population)	66.4	68.2	67.2
Age groups (study population)			
15–69	897 (63.5)	524 (57.3)	1421
70–79	515 (36.5)	391 (42.7)	906
Age standardized incidence rates per 100,000 person-years, 2013–2016 (standard error)			
Administrative district Münster	4.98 (0.27)	2.69 (0.18)	3.75 (0.16)
Germany	5.09 (0.05)	2.71 (0.03)	3.79 (0.03)

**Fig. 1:**
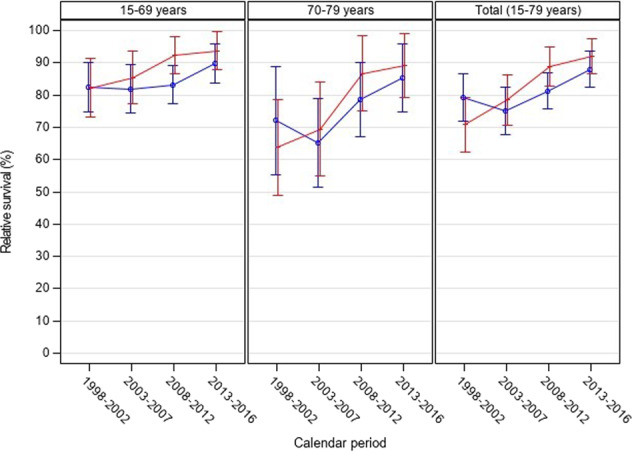
Age-standardized and age-specific relative 5-year survival for patients with CLL (administrative district of Münster, North Rhine-Westphalia, Germany). Red graphs: women, blue graphs: men, error bars indicate 95% confidence intervals (point-wise).

For men, age-standardized 5-year RS was 79.2% in 1998–2002 and 75.1% in 2003–2007 and then increased to 81.3% for the calendar period 2008–2012 and 88.0% in 2013–2016. For women age-standardized 5-year RS also increased continuously over time from 70.8% in 1998–2002 to 92.1% in 2013–2016 (78.5% in 2003–2007; 88.9% in 2008–2012). Correspondingly, age-specific trends in relative survival showed distinctly increasing rates of 5-year RS. For men aged 15–69 years 5-year RS raised about 7 percentage points from 82.5% in 1998–2002 and 81.9% in 2003–2007 to 89.8% in 2013–2016 after the introduction of the combined chemoimmunotherapy. Women aged 15–69 years showed an increase in RS almost 10 percentage points over time from 82.2% in 1998–2002 (85.4% in 2003–2007; 92.3% in 2008–2012) to 93.7% in 2013–2016. In the older age-group (70–79 years) the observed RS increase was even stronger for both, men and women. Five-year RS for 70–79-year-old men rose from 72.1% in 1998–2002 (65.1% in 2003–2007) before introduction of chemoimmunotherapy to 85.3% in 2013–2016 after introduction of chemoimmunotherapy. In older women, 5-year RS increased more steadily from below 70% (63.8% in 1998–2002; 69.4% in 2003–2007) to 86.7% in 2008–2012 and 89.1% in 2013–2016 (Fig. 1 and Table 2).

**Table 2:** Age-standardized and age-specific relative 5-year survival for patients with CLL (administrative district of Münster, North Rhine-Westphalia, Germany).

	Calendar period: relative survival (standard errors)			
	1998–2002	2003–2007	2008–2012	2013–2016
Overall	75.4 (2.9)	76.5 (2.7)	84.3 (2.1)	89.8 (2.0)
Men	79.2 (3.8)	75.1 (3.7)	81.3 (2.9)	88.0 (2.9)
Women	70.8 (4.3)	78.5 (4.0)	88.9 (3.1)	92.1 (2.8)
Age group (years)				
Men				
15–69	82.5 (3.9)	81.9 (3.8)	83.2 (3.0)	89.8 (3.1)
70–79	72.1 (8.6)	65.1 (7.0)	78.6 (5.8)	85.3 (5.3)
Women				
15–69	82.2 (4.6)	85.4 (4.2)	92.3 (3.0)	93.7 (3.0)
70–79	63.8 (7.6)	69.4 (7.4)	86.7 (6.0)	89.1 (5.0)

## Discussion

We found marked improvements in relative survival at population level subsequently to fundamental changes in first-line therapy of patients diagnosed with CLL. For both, men and women, the 5-year age-standardized RS increased about 13 percentage points after the approval of anti-CD 20 antibodies like rituximab, ofatumumab or obinutuzumab for the treatment of CLL. Results of clinical studies have shown previously that CLL patients treated with chemoimmunotherapy achieved markedly longer progression-free survival, which also translated into prolonged overall survival [6, 7]. The population-based registry data shown in this analysis confirm these findings.

Two population-based studies from Denmark and Sweden also indicated improvements in the survival of CLL patients following the introduction of chemoimmunotherapy. However, both studies analysed data up to 2013 and hence the time period studied in which chemoimmunotherapy was available was short. Further Sylvan et al. [10] analysed survival by treatment group but did not differentiate these groups into chemotherapy only and combined chemoimmunotherapy except for therapy with fluradabine, cyclophosphamide and rituximab in combination for which the longest progression-free and overall survival was observed.

With the selected method of relative survival, CLL-associated enhancements seem to be most probably associated with survival improvements shown in this work. Treatment for CLL probably is a main factor, because of the profound change of treatment options following the approval of monoclonal antibodies. Although rituximab was available prior to 2010, it was not approved for the treatment of CLL in Germany and therefore the effect on the survival of CLL patients in the earlier time periods is negligible. At this point, we cannot formally exclude that improvements in other measures such as supportive medication, or improved anti-infective therapies had a positive influence on survival. However, there were no marked changes in this patient management with regard to these health problems during the reported time period.

Unfortunately, we cannot analyse the results according to the known prognostic factors in CLL, as this data is not reported to the registry. However, the distribution of factors such as deletion 17p, TP53 mutation, deletion 11q or an unmutated IGHV status is relatively constant over time, so that the influence of these factors on the present work can most likely be neglected.

Two points make the results particularly relevant and demand further transfer of knowledge between clinical and epidemiological research and routine care. First, the improved survival rates of patients with CLL in routine care show that the knowledge gained from clinical research and the advantages of newly approved drugs are also evident in routine care. The results presented here show a marked increase of the overall survival in the era with monoclonal anti-CD20 antibodies when compared to the era before, where these antibodies were not available for CLL therapy. It must therefore remain a goal to publish relevant study results without delay and to update guidelines for routine care as quickly as possible.

On the other hand, the work also results in a gain of knowledge for the registry strategy. In order to be able to do population-based research accompanying clinical studies, it should be discussed to expand and specify the collected data sets. While data on therapies and on the course of the cancer have recently been added to cancer registration and will be available for future analyses, data on genetic prognostic factors should furthermore be included in the registry data sets.

We recognize that our analysis has some limitations. First, some of our survival estimates are based on small numbers and consequently survival is imprecisely estimated especially when we stratify for age. Second, our time trend analysis of survival assumes that the stage distribution of CLL remained constant over time. Unfortunately, data on stage (Binet) were too incomplete for a meaningful analysis of stage shifts over time. Early stage and asymptomatic CLL cases are usually diagnosed and treated in the ambulatory setting (including private practitioners) and reporting of incident CLL cases by private practitioners may be less complete. As a consequence, our report may underrepresent cases with early stages of CLL. However, the age-standardized incidence rate in the studied region is the same than that estimated for Germany and there is no indication that private practitioners’ reporting to the cancer registry gradually increased over our study period; therefore, our time trend analysis of survival would not be substantially affected by an under-registration. Third, the high DCO among patients aged 80 years or more forced us to exclude this age group and therefore our results may not be generalizable to CLL cases aged 80 years or more. Fourth, as data on therapy were too incomplete for the study period, we could not directly study the influence of the introduction of chemoimmunotherapy on survival. Our interpretations are based on an ecologic approach, which has independent confirmatory value nevertheless. Fifth, we realize that the same type of analysis now needs to be done after the introduction of targeted agents such as BTK inhibitors or venetoclax to test for the effect of these drugs on overall survival. This may take a few years until the registry data have matured enough for this analysis.

Taken together, our results suggest that chemoimmunotherapy with anti-CD20 antibodies has improved survival in CLL patients in North Rhine-Westphalia, Germany.

### Reporting summary

Further information on experimental design is available in the Nature Research Reporting Summary linked to this article.

## Supplementary information

aj_checklist_20-BCJ-0894
